# Expanding the reach of medical physics: Immunotherapy should be included as part of the curriculum for medical physics education and training

**DOI:** 10.1002/acm2.12796

**Published:** 2019-12-20

**Authors:** Narottam Lamichhane, Matthew T. Studenski, Yi Rong

**Affiliations:** ^1^ Department of Radiation Oncology The University of Maryland School of Medicine Baltimore MD USA; ^2^ Department of Radiation Oncology The University of Miami Miller School of Medicine Miami FL USA; ^3^ Department of Radiation Oncology University of California‐Davis Cancer Center Sacramento CA USA

## 1 INTRODUCTION

From the classical radiobiology course in a medical physics graduate program, we learned the most important principle in terms of cell behavior under different radiotherapy dose‐fractionation schemes: repair, reassortment, reoxygenation, and repopulation (a.k.a. “4 Rs”). The recent breakthrough in combining stereotactic body radiotherapy (SBRT) as an induction regimen for immunotherapy might suggest a “Fifth R”: radiosensitivity.^1^ So how important is it for medical physicists to have the fundamental knowledge of this new concept? Or perhaps a more relevant question is how impactful is this concept to the field of radiation oncology? One of our previous parallel opposed editorials discussed whether the immunotherapy dictates the future of SBRT.^2^ In the current issue, the discussion continues on the importance of this new emergent field to our medical physics profession. Herein, we have Dr. Narottam Lamichhane arguing for the proposition “Immunotherapy should be included as part of the curriculum” of Medical physics education and training, and Dr. Matthew Studenski arguing against.

Narottam Lamichhane, PhD, is an assistant professor and medical physicist in the Department of Radiation Oncology at the University of Maryland School of Medicine. He graduated from the Virginia Commonwealth University with PhD in Medical Physics. He completed his therapeutic medical physics residency from the University of Miami Miller School of Medicine. His training and research interest focus are on the treatment planning, quality assurance, imaging, and experimental therapeutics.

Matthew Studenski, PhD, graduated from the University of Michigan with BSE (2005) and MSE (2006) degrees in Nuclear Engineering and Radiological Sciences. He then attended the University of Florida where he completed a second MS (2008) and PhD (2009). He moved to Thomas Jefferson University where he finished his residency in Therapeutic Radiological Physics and obtained his board certification. In 2013, he moved to the University of Miami where he is currently an Associate Professor. In addition to his clinical role, he is also the Residency Director and Brachytherapy Director. He is the chair of the AAPM ROMPES subcommittee and a CAMPEP (Commission on Accreditation of Medical Physics) residency program reviewer.

## 2 OPENING STATEMENT

### 2.1 Narottam Lamichhane, PhD

Recent advances in immunotherapy have immensely impacted the field of cancer therapy. Successes of many checkpoint inhibitors and their subsequent FDA approvals for use in various cancer types have changed the outlook of cancer treatment.^3^ The emergence of immunotherapy in radiation oncology has swept the field, with multiple clinical trials which combine these modalities in various forms are listed on clincaltrials.gov. On the motion of the success of immunotherapy, efforts on immunomodulation using radiation therapy have also been attempted.^4^ Immunomodulatory agents have the potential to bolster the anti‐tumor immune responses and provide substrates for checkpoint inhibitors to act upon. This combination of radiation with immunotherapy provides a synergistic action to better enhance the therapeutic efficacy of treatment. Radiation induced DNA damage in cancer cells and the subsequent immunogenic cell death leads to release of tumor antigens, increased infiltration of effector cells, and the activation of cytotoxic T cells to reinvigorate the anti‐tumor immunity.^5^, ^6^ This unique strategy of utilizing one modality of cancer treatment to trigger the response for and overcome the resistance against another modality is intriguing and has shown immense promise.^6^ The radiation oncology physician community has taken notice of this promise, initiating studies which have been focused on understanding the determinants of success vs. failure of the combination of radiation therapy with immunotherapy. Various immunotherapies are now approved for use in cancer patients as not only secondary options to conventional therapies but also as first‐line treatment.^7^ The impact of these recent developments of cancer immunotherapy in the field of medical physics is inevitable. This ever‐changing field of immunotherapy and its impact in radiation oncology will also steer the wheel of medical physics. The medical physics community should adapt to this change by reflecting it in education and training of future medical physicists.

The landscape of radiation oncology has evolved throughout the years. With this progression, medical physics has also advanced in parallel to match the increased demand of radiation oncology. With the widespread implementation of specialized techniques such as intensity modulated radiation therapy (IMRT), stereotactic radiation therapy (SRS/SBRT), the demand for qualified medical physicists has grown profoundly. To match this need, the education and training for medical physicist pre‐ and post‐graduation has seen an unprecedented change in past few years.^8^ Along with the clinical training, the research component of medical physics has also been encouraged; hence advanced. Many medical physics graduate programs and medical physics residency programs sought out the accreditation process from CAMPEP to standardize the education and postgraduate training of medical physicists.

However, the present scenario of radiation oncology emerged so drastically; that the field no longer can be seen as singular. The care within radiation oncology has turned into a multidisciplinary approach of combining molecular imaging, immunology, medical oncology, and other specialties. This has helped tailor individualized treatment for patients, promoting a concept of precision medicine in radiation oncology. However, the education curriculum for medical physics may not have transformed to reflect the direction of the field. The use of molecular imaging such as PET/SPECT/MRI is routinely being utilized in radiation oncology. This has been reflected in medical physics curriculum where the graduate students and medical physics residents undergo courses and rotations in imaging to cover basics of all imaging modalities. Biological interactions of radiation with cells and how it affects the biology of tumor are also covered during the graduate school for medical physics and during the residency training. However, with the ever‐increasing utilization and exploration of immunotherapy in radiation oncology in past few years, understanding the basic immune system, immune checkpoint, signaling pathways, receptor/ligands will turn out to be beneficial for medical physicists. As it stands, the course work on radiobiology during the graduate school and during residency training may not prepare medical physics students and residents to the basic intricacies of tumor immunology. An excerpt of immunology in conjunction with radiobiology will be a tremendous help for medical physicists to tread in the current field of immunotherapy and its impact in radiation oncology. Increasing the communication between these two specialties will foster the infrastructure of clinical radiotherapy. Even with an advanced education program IBPRO (Integrated Course in Biology and Physics of Radiation Oncology) which was funded by NCI, the theme of immunotherapy was included in year 2016 to advance the knowledge and skill required to radiation oncologists, physicists, and biologists to promote the interdisciplinary collaboration to improve the quality of cancer care.^9^

Coined as a term “Medical Physics” for the use of physics in medicine in 1778, the field of medical physics reflects all aspects of physics in various fields of medicine.^10^ However, as rich and deep its origin is, the field is still fresh with continual room for growth, optimization, and exploration. Finding a niche in immunology remains an outstanding opportunity for medical physics. Educating medical physicists in basics of tumor microenvironment and immunology will be fruitful in overall understanding of cancer dynamics. Furthermore, given the intimate work relation of physicists and physician, this understanding will prove necessary when leading discussions related to various cancer topics not only for treatment but also for the opportunities in cancer research. The benefits of educating medical physicists on the basics of immunology and its long‐term impact on clinical, academic, or research practice of medical physics may be unclear, but as professionals who are to understand the entire radiation therapy process and its position in cancer treatment, knowledge of the basics of immunology will only benefit our field as a whole. With the tremendous efforts on immunotherapy and its significant positive clinical outcomes in various cancers,^3^, ^4^, ^5^, ^11^ the radiation oncology physician community followed this train ride. It is time for the medical physics community to hop‐on.

### 2.2 Matthew T. Studenski, PhD

When you consider the term medical physics, this is by definition, any physics‐related topic applied to medicine. With physics as the fundamental base for all the sciences, almost any science‐based topic related to medicine could be construed to fit under the umbrella of medical physics. That said, it is impossible to become an expert in such a broad field so in practice, medical physicists have become more specialized. Although the modern medical physicist may be specialized, there are still an immense set of knowledge and skills required. The question becomes, how do you educate a person to become a medical physicist and what topics should be covered?

In the past, there was no fixed path to become a medical physicist. As long as your educational background was related to physics or engineering and you had an advanced degree, once you decided to practice medical physics, on the job training was used to hone your skill set. More recently, the clinical role of medical physics has grown due in part to the introduction of new technology in the fields of radiation oncology, radiology, and nuclear medicine. As this technology can harm a patient, there needs to be assurance that medical physicists who are responsible for implementing this technology are qualified to do so. The overall consequence is that clinical medical physics has gone down the path of board certification to establish competency, similar to our physician colleagues. In the United States, the American Board of Radiology (ABR) has become the major organization that certifies clinical medical physicists and certification is now a goal as it “is the best measure of the knowledge, experience and skills needed to provide quality patient care”.^12^ Furthermore, many institutions require this certification to practice as a medical physicist.

The pursuit of certification has reshaped the educational pathway to become a practicing medical physicist. When the ABR introduced the 2012/2014 initiative, the path became very standardized; equivalent of a physics minor as an undergraduate, a fixed core curriculum as a graduate student and final clinical training as a resident, both from CAMPEP accredited institutions 8. To become accredited, CAMPEP requires that institutions have qualified faculty that teach a specific core curriculum to prepare students to enter the field of medical.^13^, ^14^, ^15^

With the clinical medical physics curriculum now defined by the path to certification, one topic not included in the curriculum is immunotherapy. Immunotherapy is “A type of cancer treatment that helps your immune system fight cancer. It is a type of biological therapy. Biological therapy is a type of therapy that uses substances from living organisms to treat cancer”.^16^ Based on this definition, immunotherapy is not a physics topic and therefore should not be included in the medical physics curriculum. On the other hand, since biology at its core can be related to physics topics, maybe the topic of immunotherapy does fall under the umbrella of medical physics. Assuming immunotherapy is a medical physics topic, there are still several other reasons that it should not become part of the medical physics curriculum.

First, the medical physics curriculum is already extensive. If you consider the residency curriculum required by CAMPEP,^15^ it is already difficult to include all the clinical topics into a 2‐year residency and ensure the resident obtains enough practical training to become proficient. The goal of a residency is to train competent clinical medical physicists and the focus should be on clinical topics that residents could be responsible to cover in the future. You could go further and argue that the curriculum is already outdated and lacking some of the more modern modalities that are physics related (e.g., proton therapy). If a topic is to be added, it makes more sense to add a physics topic like proton therapy than a biology‐based topic like immunotherapy.

Second, if immunotherapy was added to the medical physics curriculum, who would instruct this course? In a larger institution, there is a possibility that a faculty member with immunotherapy experience would be available. In smaller institutions, this responsibility would fall to the physicists who may or may not have any immunotherapy training or experience. One of the important characteristics of an effective teacher is to be prepared, which is difficult when a topic is unfamiliar.^17^

Finally, one could argue that throughout the course of a medical physics graduate degree and residency program, one would be exposed to the topic of immunotherapy at some point during grand rounds or other clinical lectures. If that is not enough depth because a student is very interested in immunotherapy, they always have the option of self‐directed study. Although immunotherapy is becoming more commonplace in cancer treatments, it is a biology‐based topic that should not be added into the current medical physics curriculum. The current curriculum is already extensive and possibly lacking physics‐based topics related to emerging technology.

## 3 REBUTTAL

### 3.1 Narottam Lamichhane, PhD

Healthcare field, and especially patient care, has become increasingly an interprofessional unit. With the advent of various treatment modalities and research demonstrating the interdependence of these modalities in the treatment outcomes, it is essential for medical physicists to have a basic understanding of complementary treatment modalities that improve efficacies of radiation therapies and the biology behind the clinical outcomes of these combinations. Learning is a lifelong process, and as medical physicists we owe it to our patients to have a basic understanding and explore the potentials of new treatment modalities, such as immunotherapies, when combined with radiation therapy. I agree with Dr. Studenski that the course load and certification requirements for medical physics require rigorous efforts from physicists. Additionally, it is true that new technologies in radiation oncology physics will require additional courses aimed at educating future physicists about these new technologies. It is, however, short sighted to ignore the importance of an immunology course in the training of medical physicists. After all, the outcomes of radiation treatments depend on biological changes (cellular and molecular); and recent evidences demonstrate that a major contributor to the outcomes is the activation of the host immune responses.^5^ In order for one to be able to improve on, troubleshoot, and evaluate a rationale combination of immunotherapies with radiation therapy, it is imperative that one understands the immunological concept behind the outcomes of these treatments. In recent years, there have been a flurry of research publications and clinical trials evaluating the efficacies and biological inferences of combination of radiation therapies with immunotherapies against various cancers. With these developments, it is not far reaching to expect that in many cases, treatment of cancers with radiation modalities will certainly involve combination with some form of immunotherapy. In this regard, medical physicists without a background in basic immunology will be at a disadvantage in both the clinical care of the patients as well as in exploring cutting edge research in this field. While it is impossible to train student physicists in every aspect of immunology and immunotherapy, a basic course in immunology will certainly provide a basic foundation for physicists to understand and explore the biology, pros, and cons of radiation therapy’s combination with immunotherapies. A graduate level basic immunology course does not require many additional credit hours. A conjunction of this course with radiation biology will not only bolster the course but also pave a way for medical physicists to navigate in a diverse biological dimension. Additionally, most institutions that offer a degree in medical physics are multidisciplinary; hence finding a trained faculty to offer this course should not be an impediment. Without a course in basic immunology, understanding of immunotherapies and especially understanding the immunological landscape of combination therapies will be superficial. As demanding as medical physics curriculum is, we cannot ignore the importance of education in basic immunology in light of the multidisciplinary approach in delivering combination treatments, role of immune system and its modulation in clinical outcomes of radiation, radiation + immunotherapies, and cutting edge research aimed at improving efficacies of different radiation modalities through combinations. Therefore, addition of immunology course in medical physics curriculum has more long‐term pros than short‐term cons.

### 3.2 Matthew T. Studenski, PhD

Dr. Lamichhane has made a compelling argument for the emergence of immunotherapy and its potential for integration into radiation therapy in the future. He also made a strong point that it is essential for medical physicists to have many tools in their arsenal as healthcare is becoming more interdisciplinary. That said, the question here is not to determine whether immunotherapy is important, it is to determine whether the medical physics core curriculum should be modified to include a course on immunotherapy. Furthermore, we need to consider if it is possible for a physicist to have every imaginable tool in their belt.

Dr. Lamichhane stated that the implementation of IMRT and SBRT resulted in a change in the core medical physics curriculum and immunotherapy should follow suit. It is not clear that this comparison is fair as immunotherapy is biology‐based while IMRT and SBRT are definitely physics‐based and require medical physicist involvement to ensure proper delivery. Currently, immunotherapy drugs simply enhance the effect of the radiation damage and do not require medical physics intervention beyond what is already in place for standard linear accelerator QA. Unless physicists were to become more involved in the biological and chemical aspects of immunotherapy delivery, a course addressing this topic is not warranted.

If we were to modify the medical physics core curriculum to include a course on immunotherapy, there are hurdles and logistical issues that need addressing similar to those noted in a previous point‐counterpoint discussion.^18^ The first hurdle is the addition of credit hours. Students (especially those pursuing a master degree) already have a full course load. Additional credit hours incur both a time commitment and a financial commitment that many students might not be able to accommodate. The other option would be to eliminate or reduce the length of courses in the current curriculum. There is no doubt that some of the material in the current curriculum is outdated, but it would be difficult to eliminate enough to allow the addition of a full course. This would potentially exclude new physics‐based technologies such as MRI‐linac or proton therapy, which are not part of the curriculum either. The second hurdle is finding qualified instructors for the immunotherapy course. Dr. Lamichhane suggests that if there is not a member of the medical physics faculty who is an expert in immunotherapy, one could simply find another faculty in a different department who could teach the class. Although possible, many universities do have limitations that could make this a difficult task.

On the other hand, inclusion of immunotherapy as a smaller component of a clinical rotation or course is a possibility; think Y90 microspheres. Y90 is also a multidisciplinary treatment but it is our responsibility as physicists to understand the dose deposition and radiation physics, not the process of implanting a catheter in the liver close to the tumor. Because of this, Y90 microspheres are included as part of the core curriculum but in limited detail (i.e., one lecture in a radiotherapy course or one part of a larger brachytherapy rotation during residency). This would also reduce the pressure to find faculty who have a deep understanding of immunotherapy. An approach adopting one or two lectures on immunotherapy is a possibility, but devoting more time than that would not be appropriate.

Finally, in the current medical physics curriculum, there is nothing to prevent students from taking a course on immunotherapy in another department if this is a topic of interest. Moreover, discussions on immunotherapy are likely to appear in clinical conferences and lectures throughout the student’s education. This is another opportunity for a student to investigate this topic in greater depth. If included at all in the core medical physics curriculum at all, immunotherapy should only be a small component, not a full course.
